# Understanding Test Takers' Choices in a Self-Adapted Test: A Hidden Markov Modeling of Process Data

**DOI:** 10.3389/fpsyg.2019.00083

**Published:** 2019-02-06

**Authors:** Meirav Arieli-Attali, Lu Ou, Vanessa R. Simmering

**Affiliations:** ^1^Department of Psychology, Fordham University, New York, NY, United States; ^2^ACTNext, ACT Inc., Iowa City, IA, United States

**Keywords:** hidden Markov model, self-adapted test, likelihood ratio test, goal orientation, confidence

## Abstract

With the rise of more interactive assessments, such as simulation- and game-based assessment, process data are available to learn about students' cognitive processes as well as motivational aspects. Since process data can be complicated due to interdependencies in time, our traditional psychometric models may not necessarily fit, and we need to look for additional ways to analyze such data. In this study, we draw process data from a study on self-adapted test under different goal conditions (Arieli-Attali, 2016) and use hidden Markov models to learn about test takers' choice making behavior. Self-adapted test is designed to allow test takers to choose the level of difficulty of the items they receive. The data includes test results from two conditions of goal orientation (performance goal and learning goal), as well as confidence ratings on each question. We show that using HMM we can learn about transition probabilities from one state to another as dependent on the goal orientation, the accumulated score and accumulated confidence, and the interactions therein. The implications of such insights are discussed.

## 1. Introduction

With the rise of interactive assessment and learning programs, process data become available to infer about students' cognitive and motivational aspects. Process data can help us learn about students' strategies, preferences, and attitudes. In the context of problem solving, detecting strategies may reveal the cognitive processes needed to perform the task, and may even be considered as a factor in ability estimating (DiCerbo and Behrens, 2012; Liu et al., 2018). However, interactive assessments such as simulation- and game-based assessments often afford opportunities to make choices about the course of game/simulation (e.g., which variables to try in the simulation, which path to take in the game) that are not directly connected to ability albeit may influence its assessment. Such choices may be a result of or reflect metacognitive or motivational aspects of task performance. For example, students' self-estimated knowledge and belief in their ability, students' tendency toward challenge, or whether students are motivated to do their best or just perform at minimum effort are just a few of the factors that may play a role in choices made in interactive assessment.

Metacognition of task performance is rarely assessed as part of educational or academic assessments, yet it is acknowledged as important in student performance (Camara et al., 2015). One aspect of metacognition is the Feeling of Knowledge (FOK; Koriat, 1993) that is evoked naturally when attempting to answer a question. The cognitive process of attempting to answer a question evokes the FOK based on the implicit and explicit accessibility cues (the easiness of accessing the answer, the vividness of the clues, the amount of information activated, etc.), and the content of that knowledge, its coherence, and the inferences that can be made from various clues retrieved (cf. Koriat, 1993, 2000). The more information activated and the easier it is accessed, the more confident a person is in his or her answer. Asking people to evaluate their level of confidence in answering a question is the most common way to eliciting their FOK estimation and is a moderately valid predictor of actual knowledge (Koriat, 1993, 2000; Wright and Ayton, 1994).

Feeling of knowing and estimation of one's own ability relate to and affect a student's engagement or motivation when performing a task, which is called the “expectancy component” in the Expectancy-Value Model of motivation by Pintrich and colleagues (Pintrich, 1988; Pintrich and De Groot, 1990; Pintrich and Schunk, 2002). Another component of the Expectancy-Value Model is the perceived value of the task. One aspect of perceived value is the goal orientation toward the task. Research on goal orientation of task performance yields a primary distinction between “performance” and “learning” goals (Dweck and Leggett, 1988). Individuals with a performance goal strive to perform at their best to demonstrate their skills to themselves or others, while individuals with a learning goal toward a task strive to learn from the task caring less about demonstrating their skills. Although individuals often exhibit these attitudes in general (Dweck et al., 1995), studies have shown that the orientation goal can be changed via psychological intervention given prior to performing a task and even only by the instructions of the task (Dweck, 2006). One of the pervasive findings regarding this distinction is that students with a learning goal are more motivated and seek more challenges (Dweck, 2006; Blackwell et al., 2007; Yeager and Dweck, 2012).

In this study we tap into motivational and metacognitive aspects of task performance via modeling process data. We are analyzing data from a previous study (Arieli-Attali, 2016) that applied the goal-orientation manipulation in a self-adapted test, while collecting also confidence ratings. Self-adapted testing is designed to allow test takers to choose the level of the difficulty of the items they receive. In her study, Arieli-Attali (2016) instructed participants in one condition to perform at their best on the test, with incentive of a reward; participants in the second condition were instructed to use the self-adapted test as a learning tool for a test the following day. Main findings showed that participants in the learning goal condition chose overall more difficult items (about half a level on average out of seven possible levels) compared to the performance goal condition, after controlling for pre-test performance, manifested both in the start of the test (the first choice) and the mean choices across all items. In addition, participants in the learning goal condition reverted to a strategy of choosing only the easiest level for all items significantly less frequently than those in the performance goal condition did (3.4% compared to 11.5%, respectively), and showed more exploratory behavior by choosing a wider range of difficulty levels (range of 3 levels compared to 2.5 levels in the performance goal condition).These results support the general theory and converge with previous findings by Dweck and colleagues about the higher motivation and tendency to seek more challenges when one is holding a learning goal orientation. Regarding confidence ratings, Arieli-Attali found that those in the learning goal condition showed under-confidence while those in the performance goal condition showed over-confidence (− 1.4 vs. +1.9% respectively), similar to a recent study by Dweck and colleagues (Ehrlinger et al., 2016). Using the process data from Arieli-attali's study will allow us to tap deeper into the dynamics of choices as changing over time and depending on goal orientation and confidence rating. Before we describe the details of the current study, we provide a brief summary of research on self-adapted testing.

Self-adapted tests are designed to allow test takers to choose the level of difficulty of the items they receive (Rocklin and O'Donnell, 1987; Wise et al., 1992; Hontangas et al., 2004; Arieli-Attali, 2016). Such tests provide both product data—which items were answered correctly—as well as process data—what difficulty levels were chosen across time. Using an item response theory modeling approach, each test taker's ability can be estimated using the product data regardless of the item difficulty levels chosen. However, the difficulty preferences (the process data) may also be useful as an indication of the test taker's metacognitive and/or motivational state.

Previous studies on self-adapted tests were primarily concerned with the product data and its reliability and validity. However, there were also studies that looked into the process data particularly to examine the strategies of test takers in choosing the difficulty levels (Rocklin, 1989; Johnson et al., 1991; Ponsoda et al., 1997; Hontangas et al., 2000; Revuelta, 2004). In these studies, strategies were examined with regards to correct or incorrect responses to the adjacent preceding item, based on the assumption that the “results” on a previous item, whether correct or incorrect, would affect the next choice. Researchers were interested in uncovering the “rules,” if existed, in examinees' choices, mostly adopting the approach of defining predetermined rules and looking in the data to find them. For example, Rocklin (1989) defined a “flexible strategy” as a selection of an easier level after an incorrect response, and a more difficult level after a correct response. This strategy is intuitive and in fact simulates the sequence of item difficulty produced by a Computer Adaptive Test (CAT) algorithm that maximizes test accuracy, where test takers often receive an easier item after incorrect response, and a harder item after a correct response, based on item response theory (Hambleton and Swaminathan, 1985). Defining such a strategy is based on the intuition that this would also be the most “rational” strategy people are using in their choices. In addition to the flexible strategy, Rocklin (1989) defined two variations: the “failure tolerant” and “failure intolerant.” In the former, selections do not change after incorrect response (thus, showing tolerance to incorrect/failure), and in the latter, selections do not change after correct responses. Findings from this study and another study that followed (Johnson et al., 1991) showed that few test takers adhere to one of the three clear-cut categories, while most people exhibit more of a mixed strategy (or what Johnson et al., 1991 termed as “sluggishly flexible”) where test takers selected a harder level after one or a string of several correct responses, and selected an easier level after one or a string of several incorrect responses. In other studies (e.g., Hontangas et al., 2000; Revuelta, 2004) authors made somewhat different distinctions (such as totally rigid, partly flexible, and partly rigid); however, the findings were still very similar, showing that the majority of test takers are in the “partly rigid partly flexible” category, supporting previous findings. In Revuelta (2004)'s study, the author also reported that a majority of selections (about 60%) had the same difficulty level as the previous item.

In the current study, we take a different approach to look at the sequences of difficulty choices. Although we still look at transitions, we adopt a hidden latent approach rather than direct analysis of the observed choices. In addition, due to the inter-dependencies among difficulty choices, we apply a hidden Markov model (HMM). Under an HMM we assume independence between the observed choices conditional on respective latent states, which follow a first-order Markov process such that the current state only depends on the previous state. We explain initial states and state transitions in terms of probabilities and the effects of covariates on these probabilities. The HMM approach, as well as other variations of Markov models, are becoming increasingly popular among the educational measurement community for cognitive modeling (Yudelson et al., 2013; Li et al., 2016; LaMar, 2018; Wang et al., 2018) and analyses involving serially dependent process data (Vermunt et al., 1999; Dutilh et al., 2010; Bergner et al., 2017; Shu et al., 2017). We add to the literature an application of the HMM approach in characterizing test takers' behavior in self-adapted tests. The advantages of using this approach in our context are three-fold: (1) the introduction of the latent state as the metacognitive and/or motivational state that drives the observed difficulty choices can separate the stochasticity in the underlying metacognitive process from measurement errors; (2) it allows the same observed difficulty level to be a reflection of different latent states depending on the choices before and after (see **Figure 5** below for a specific example); (3) the estimation is robust against some design decisions such as the number of difficulty levels offered in different applications of self-adapted testing (whether 5, 7, or 9 difficulty levels are offered may change the observed sequence).

## 2. The Current Study

In this paper we conduct a secondary analysis of the data from Arieli-Attali (2016). The original study evaluated how the goal orientation conditions affected test takers' item difficulty choices, as well as the influence of different feedback conditions that will not be considered here. The aim of the current analysis is to model test takers' choices of item difficulty under the two orientation goal conditions, while taking into account the correctness and confidence ratings of previous items. We applied a first order Markov process, that looked at the change of the current state/class as dependent on the previous one. However, we used accumulated correctness and confidence as predictors. That is, we assumed that accumulated prior results of overall success (accumulated correct answers) and overall state of FOK (accumulated confidence) would affect the latent state and hence the next observed choice.

Using HMM we obtained the transition probabilities between the latent classes. Transition from a class with lower difficulty level to one with a higher difficulty level (i.e., an upward transition) represents a scenario where a test taker was willing to take on higher difficulty levels presumably due to increase in motivation, openness to challenge and exploration and/or increase in self-perceived ability due to evidence of success. On the contrary, a transition from choosing higher to lower difficulty items (i.e., a downward transition) illustrates the case where a test taker preferred to lower the difficulty, presumably due to a decrease in motivation or to alleviate stress, and/or as a strategy to get a better score/feedback (get more items correct).

Our first research question concerned modeling the transitions between latent states given the current state in the two goal conditions. Based on Arieli-Attali (2016)' results we anticipated that participants in the performance goal condition would not only have higher probability of choosing the lower difficulty state initially but also transition less from this state.

Our second research question addressed transitions in difficulty as dependent on correctness of and confidence on past items responses. We hypothesized that overall accumulated correctness and confidence would interact such that being correct and confident would generally enhance upward transitions while being incorrect and unconfident would enhance downward transitions. Regarding transitions in the mis-match cases of being correct with low confidence (under-confident) or being incorrect with high confidence (over-confidence), we hypothesized overall more transitions in both directions resulting from the conflict between confidence and feedback about correctness.

The paper is organized as follows: we first describe the data and the modeling approach. Next we provide some insights into the data using visualization of the raw data, the most common sequences and the patterns observed. We then report the results of the HMM analysis addressing specifically the two research questions. Lastly, we discuss these results in relation to their contribution to the emerging field of analyzing process data in assessment.

## 3. Methods

### 3.1. Participants, Design, and Procedure

Arieli-Attali (2016) reported a final sample of 583 adult participants (age range = 18–74 years, *M* = 33.09; 45% women), recruited through Amazon Mechanical Turk (limited to native English speakers and residents of the US or Canada), who participated in a task over 2 days. Ethics approval for the study was obtained from Fordham University Institutional Review Board and a written informed consent was obtained from all participants (for the IRB approval and informed consent form see appendix E in Arieli-Attali, 2016). Our analysis includes data only from Day 1 of the experiment. On Day 1, participants completed a 24-item non-adaptive pre-test and a 40-item self-adapted test, both comprising open-ended general knowledge items. We used the pre-test scores that were obtained in the form of percentage of correct responses (ranged from 0.22 to 1, with a mean of 0.75, and standard deviation of 0.16). Following completion of the pre-test, participants were randomly assigned to one of two goal conditions: 286 participants were in the performance goal condition (condition = 1), instructed to maximize their score on the test, and 297 were in the learning goal condition (condition = 0), instructed to use the test as a learning tool for the test the next day. During the self-adapted test, participants chose a difficulty level for each item out of seven difficulty levels offered. After responding to each question, participants rated their confidence in their answer on a scale from 0 to 100 with 10-point intervals. After submission of the answer and the confidence ratings, participants received feedback whether their answer was correct or not and were provided with the correct answer. Coding of correctness was 0 for incorrect and 1 for correct. The observed item difficulty levels were integers from 1 to 7, which we divided by 7 to arrive at a range comparable with other variables used in the model fitting. Confidence reporting was converted proportionally to a scale from 0 to 1.

### 3.2. Modeling

**Figure 1:**
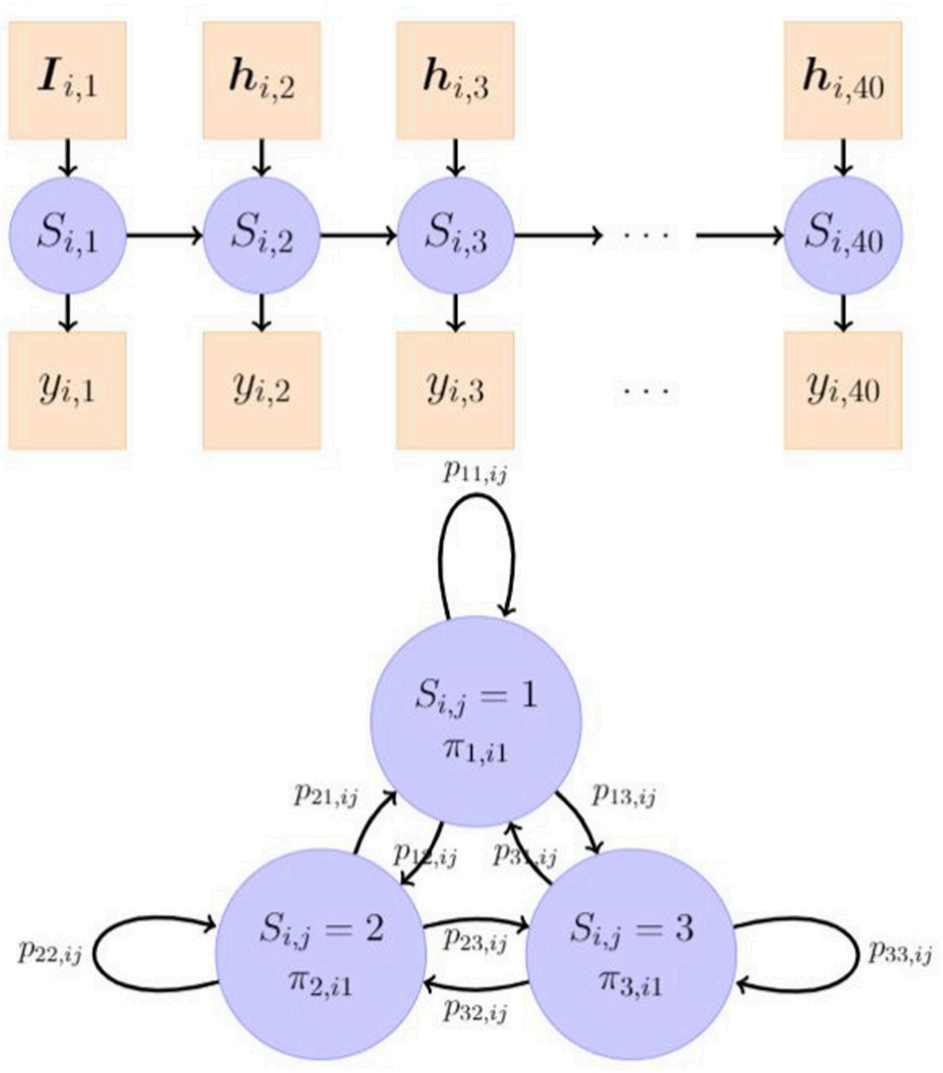
An illustration of a 3-state hidden Markov model. The latent categorical *S*_*i,j*_ is linked to the observed variable *y*_*i,j*_, *j* = 1, 2, ⋯ , 40 through a measurement model. π_*m,i*1_, *m* = 1, 2, 3 is the probability of individual *i*'s being initially in the class *m* and is explained by observed covariates ***I***_*i,j*_. *p*_*lm,ij*_ is the probability of individual *i*'s transitioning from class *l* at item *j* − 1 to class *m* at item *j*, and is explained by observed covariates ***h***_*i,j*_.

We modeled test takers' choices of item difficulty using a hidden Markov model (HMM; Vermunt et al., 1999; Böckenholt, 2005; Visser and Speekenbrink, 2010; Visser, 2011) that assumed the manifest variables (i.e., item difficulty choices) are conditionally independent given an underlying latent Markov chain with a finite number of latent states or classes of the general difficulty preferences. We assumed that there are *M* states in the Markov chain. In the following text, we use “state” and “class” interchangeably to refer to the latent state of the *M*-state Markov chain, which is denoted as *S*_*i,j*_, where integers *i* and *j*, respectively index participants and items. The categorical variable *S*_*i,j*_ was an integer element from the finite set {1, 2, ⋯, *M*} and varies across people and items. In our measurement model (as shown in the upper panel of Figure 1), we assumed that the conditional distribution of the manifest choices of item difficulty, *y*_*i,j*_, given *S*_*i,j*_, was univariate normal with mean μ_*S*_*i,j*__ and variance of $\sigma_{S_{i,j}}^{2}$. Although *y*_*i,j*_ was ordinal in our current study, we treated it as continuous because we conceptualized the 7 manifest difficulty levels as a continuum representing participants' preferences of item difficulty and the intervals between any two points were approximately equal. The seven-level difficulty structure corresponded to the seven categories of a categorized item difficulty continuous scale (− 3, − 2, − 1, 0, 1, 2, 3). The average difficulties of items at each difficulty level are: − 3.3, − 1.8, − 0.9. − 0.2, 0.5, 1.0, and 1.8 for level 1 through 7 respectively (corresponding to 92, 80, 68, 55, 41, 30, and 16% average probability of correct answer at each level) (Arieli-Attali, 2016). So the data were an ordinal approximation of a continuous variable. Practically, the rule of thumb is that ordinal variables with five or more categories can often be used as continuous without substantial harm to the analysis (Johnson and Creech, 1983; Norman, 2010; Rhemtulla et al., 2012). There were 7 categories in our study. We preferred to treat the data as continuous rather than as categorical for ease of interpretation. Depending on the magnitude of μ_*S*_*i,j*__, each class thus represented a more general item difficulty level that the participants feel comfortable choosing but may stochastically end at different manifest choices according to the measurement model.

In the latent model (as shown in Figure 1), we assumed that the change process of *S*_*i,j*_ followed a first-order Markov chain process, where the current state only depended on the previous state. We described the dynamics of *S*_*i,j*_ through its initial state and transitions between the states. The former depends on a *M*×1 vector of initial state probabilities, **π**_*i*1_ = [π_*m,i*1_], and the latter is characterized by a *M*×*M* matrix of transition probabilities of moving from a state *l* to a state *m*, ***P***_*ij*_ = [*p*_*lm,ij*_], whose *k*-th row is denoted as ***P***_*ij,k*_. Individual differences in the dynamic processes of *S*_*i,j*_ were assumed to lie in the initial state probabilities and the transition probabilities, represented by two multinomial logistic regression models as follows:

(1) $$\mathrm{Pr}(S_{i,1}=m|I_{i,1})≜\pi_{m,i1}=\frac{\exp(a_{m}+b_{m}^{T}I_{i,1})}{\sum_{k=1}^{M}\exp(a_{k}+b_{k}^{T}I_{i,1})},$$

(2) $$\mathrm{Pr}(S_{i,j}=m|S_{i,j-1}=l,h_{i,j})≜p_{lm,ij}=\frac{\exp(c_{lm}+d_{lm}^{T}h_{i,j})}{\sum_{k=1}^{M}\exp(c_{lk}+d_{lk}^{T}h_{i,j})},$$

where *m* = 1, 2, ⋯ , *M* denotes the latent classes, ***I***_*i*,1_, ***h***_*i,j*_ are vectors of covariates used for prediction in the logistic regressions, *a*_*m*_ and *c*_*lm*_ denote the logit intercepts, and ***b***_*m*_, and ***d***_*lm*_ denote the regression coefficients of the covariates in the associated log-odds (LO) relative to a specified reference class. In the current study, we predicted the initial class probabilities, π_*m,i*1_, using the goal condition (abbreviated as *d*), pre-test score (abbreviated as *p*), and their interactions, and explain the transition probabilities, *p*_*lm,ij*_, using the goal condition, accumulated correctness (abbreviated as *r*), accumulated confidence (abbreviated as *f*), and the interactions therein. The accumulated correctness and confidence at item *j* were calculated as the percentage of correctness or average confidence among items from the beginning to item *j*.

For identification purposes, both Equations (1) and (2) require specification of a reference class where all parameters in the regression equation are zero, which ensures that the initial class probabilities across all classes and the probability of moving into any class from a single class sum to 1.0. π_*m,i*1_ is the probability of individual *i*'s being initially in the class *m*, and the regression coefficients ***b***_*m*_ denote the effects of the covariates in the LO of being initially in the class *m* relative to the reference class. *p*_*lm,ij*_ is the probability of individual *i*'s transitioning from class *l* at item *j* − 1 to class *m* at item *j*, and the slopes in ***d***_*lm*_ represent the effects of the covariates on the LO of transitioning from the *l*th class into the *m*th class relative to transitioning into the reference class. The choice of the reference class will only affect the logit regression parameters to be estimated, but will not influence the fit indices, the other parameter estimates, and the transformed estimated probabilities by a notable significant amount. Theoretically, the probability of being in the reference class cannot be zero in the model. Practically, it is recommended to choose a class that is presumably large enough and can make interpretation of results easier, for example, the normative class, the largest class, or the intermediate class. In this study, we used the default latent reference class of the R package depmixS4 (i.e., the first class), which turned out to be the medium class based on its mean estimate, but the findings should not be sensitive to this choice.

We can summarize Equations (1) and (2) into vector forms of **π**_*i*1_ = $g\left(\left[a_{m}+b_{m}^{T}I_{i,1}\right]\right)$ and ***P***_*ij*_ = $g\left(\left[c_{lm}+d_{lm}^{T}h_{i,j}\right]\right)$, where *g*(·) is the softmax (normalized exponential) function. In our full model (also shown in Table 1), ***I***_*i*,1_ is a 3 × 1 vector of the covariates *d*, *p*, and their interaction *dp*, and ***h***_*i,j*_ is a 7 × 1 vector of the covariates including *d*, *r*, *f*, three two-way interactions (*df*, *dr*, and *fr*), and one three-way interaction (*dfr*). Accordingly, there are 3 parameters in ***b***_*m*_ and 7 parameters in ***d***_*lm*_. Altogether, there are 2*M* + 4(*M* − 1) + 8*M*(*M* − 1) parameters in the model, consisting of 2*M* parameters in the measurement model, (3 + 1) parameters each for *M* − 1 regressions of initial class probabilities, and (7 + 1) parameters each (i.e., *c*_*lm*_ and ***d***_*lm*_) for *M*(*M* − 1) regressions of *M*(*M* − 1) transition probabilities.

**Table 1:** Fit indices and parameter estimates across fitted models.

**Model**	**A**	**B**	**B1**	**B2a**	**B2b**	**B3**
M	2	3	3	3	3	3
[1, **I**_*i,j*_]			[1, d, p, dp]	[1, d, p, dp]	[1, d, p, dp]	[1, d, p, dp]
[1, **h**_*i,j*_]				[1, d]	[1, f, r, fr]	[1, d, f, r, fr, df, dr, dfr]
AIC	−16658.95	−27223.54	−27282.80	−27306.69	−27466.63	−27487.31
BIC	−16602.55	−27110.74	−27121.66	−27097.20	−27160.46	−26987.77
df	7	14	20	26	38	62
logLik	8336.474	13625.771	13661.399	13679.344	13771.316	13805.653
LRT			B ⊂ B1	B1 ⊂ B2a	B1 ⊂ B2b	B2b ⊂B3
$\Delta_{\chi^{2}}\left[\Delta_{df}\right]$			71.26^*^[6]	35.89^*^[6]	219.83^*^[18]	68.67^*^[24]
μ_1_(σ_1_)	0.19 (0.07)	0.19 (0.07)	0.19 (0.07)	0.19 (0.07)	0.19 (0.07)	0.19 (0.07)
μ_2_(σ_2_)	0.62 (0.22)	0.51 (0.12)	0.51 (0.12)	0.51(0.12)	0.51(0.11)	0.51(0.11)
μ_3_(σ_3_)		0.86 (0.13)	0.86 (0.13)	0.86 (0.13)	0.86 (0.13)	0.86 (0.13)
**π**_*i*1_	$\left[\begin{array}{c} 0.33\\ 0.67 \end{array}\right]$	$\left[\begin{array}{c} 0.36\\ 0.45\\ 0.19 \end{array}\right]$	$g({\left[\begin{array}{c} 1\\ \text{d}\\ \text{p}\\ \text{dp} \end{array}\right]}^{T}\left[\begin{array}{ccc} 1.74 & 0 & -3.21\\ 0.05 & 0 & -2.29\\ -3.06 & 0 & 3.15\\ 0.6 & 0 & 2.56 \end{array}\right])$	$g({\left[\begin{array}{c} 1\\ \text{d}\\ \text{p}\\ \text{dp} \end{array}\right]}^{T}\left[\begin{array}{ccc} 1.77 & 0 & -3.16\\ -0.01 & 0 & -2.39\\ -3.07 & 0 & 3.1\\ 0.62 & 0 & 2.68 \end{array}\right])$	$g({\left[\begin{array}{c} 1\\ \text{d}\\ \text{p}\\ \text{dp} \end{array}\right]}^{T}\left[\begin{array}{ccc} 1.75 & 0 & -3.13\\ 0.03 & 0 & -2.16\\ -3.11 & 0 & 3.04\\ 0.62 & 0 & 2.35 \end{array}\right])$	$g({\left[\begin{array}{c} 1\\ \text{d}\\ \text{p}\\ \text{dp} \end{array}\right]}^{T}\left[\begin{array}{ccc} 1.75 & 0 & -3.14\\ 0.08 & 0 & -1.99\\ -3.07 & 0 & 3.07\\ 0.49 & 0 & 2.1 \end{array}\right])$
**P**_*ij*,1_	$\left[\begin{array}{c} 0.96\\ 0.04 \end{array}\right]$	$\left[\begin{array}{c} 0.93\\ 0.05\\ 0.02 \end{array}\right]$	$\left[\begin{array}{c} 0.93\\ 0.05\\ 0.02 \end{array}\right]$	$g(\left[\begin{array}{c} 1\\ \text{d} \end{array}\right]\left[\begin{array}{ccc} 2.54 & 0 & -1.13\\ 0.6 & 0 & 0.21 \end{array}\right])$	$g({\left[\begin{array}{c} 1\\ \text{f}\\ \text{r}\\ \text{fr} \end{array}\right]}^{T}\left[\begin{array}{ccc} 2.96 & 0 & -1.35\\ -2.95 & 0 & 1.33\\ -0.52 & 0 & -0.66\\ 4.12 & 0 & -0.33 \end{array}\right])$	$g({\left[\begin{array}{c} 1\\ \text{d}\\ \text{f}\\ \text{r}\\ \text{fr}\\ \text{df}\\ \text{dr}\\ \text{dfr} \end{array}\right]}^{T}\left[\begin{array}{ccc} 1.77 & 0 & -1.33\\ 3.67 & 0 & -1.04\\ -1.32 & 0 & 1.98\\ 1.6 & 0 & -0.42\\ 1.01 & 0 & -1.87\\ -4.65 & 0 & -0.07\\ -5.9 & 0 & 0.89\\ 7.92 & 0 & 1.28 \end{array}\right])$
**P**_*ij*, 2_	$\left[\begin{array}{c} 0.02\\ 0.98 \end{array}\right]$	$\left[\begin{array}{c} 0.04\\ 0.92\\ 0.04 \end{array}\right]$	$\left[\begin{array}{c} 0.04\\ 0.92\\ 0.04 \end{array}\right]$	$g({\left[\begin{array}{c} 1\\ \text{d} \end{array}\right]}^{T}\left[\begin{array}{ccc} -3.17 & 0 & -2.96\\ -0.05 & 0 & -0.19 \end{array}\right])$	$g({\left[\begin{array}{c} 1\\ \text{f}\\ \text{r}\\ \text{fr} \end{array}\right]}^{T}\left[\begin{array}{ccc} -1.21 & 0 & -3.3\\ -1.9 & 0 & 0.84\\ -4.12 & 0 & -1.37\\ 4.41 & 0 & 1.42 \end{array}\right])$	$g({\left[\begin{array}{c} 1\\ \text{d}\\ \text{f}\\ \text{r}\\ \text{fr}\\ \text{df}\\ \text{dr}\\ \text{dfr} \end{array}\right]}^{T}\left[\begin{array}{ccc} -0.91 & 0 & -3.32\\ -0.94 & 0 & 0.06\\ -1.9 & 0 & 1.77\\ -5.33 & 0 & -1.71\\ 5.38 & 0 & 0.83\\ 0.58 & 0 & -2.59\\ 2.81 & 0 & 0.66\\ -2.63 & 0 & 2.1 \end{array}\right])$
**P**_*ij*, 3_		$\left[\begin{array}{c} 0.04\\ 0.06\\ 0.9 \end{array}\right]$	$\left[\begin{array}{c} 0.04\\ 0.06\\ 0.9 \end{array}\right]$	$g({\left[\begin{array}{c} 1\\ \text{d} \end{array}\right]}^{T}\left[\begin{array}{ccc} -0.39 & 0 & 2.74\\ -0.16 & 0 & 0 \end{array}\right])$	$g({\left[\begin{array}{c} 1\\ \text{f}\\ \text{r}\\ \text{fr} \end{array}\right]}^{T}\left[\begin{array}{ccc} 0.24 & 0 & 2.93\\ -0.96 & 0 & -1.59\\ -2.36 & 0 & -0.04\\ 3.31 & 0 & 2.03 \end{array}\right])$	$g({\left[\begin{array}{c} 1\\ \text{d}\\ \text{f}\\ \text{r}\\ \text{fr}\\ \text{df}\\ \text{dr}\\ \text{dfr} \end{array}\right]}^{T}\left[\begin{array}{ccc} 0.91 & 0 & 3.43\\ -2.56 & 0 & -1.58\\ -2.13 & 0 & -3.36\\ -3.65 & 0 & -0.11\\ 5.48 & 0 & 3.78\\ 4.08 & 0 & 4.82\\ 4.3 & 0 & 0.71\\ -6.77 & 0 & -4.79 \end{array}\right])$

* *p < 0.05; d, condition; p, pre-test score; f, accumulated mean confidence; r, accumulated mean correctness*.

Parameters of the model can be estimated using the expectation-maximization (EM) algorithm, where the expectation of the complete log-likelihood function of the parameters given the observations *y*_*i,j*_ and states *S*_*i,j*_ are iteratively maximized to yield parameter estimates. In the R package depmixS4 (Visser and Speekenbrink, 2010), the EM algorithm has been implemented for unconstrained models, using the standard glm routine and the nnet.default routine in the nnet package (Venables and Ripley, 2002) in the maximization step for maximizing different parts of the expectations obtained in the expectation step. For more information on the estimation, we direct the readers to check the Visser and Speekenbrink (2010) paper.

Model fit of hidden Markov models can be compared using Akaike information criterion (AIC; Akaike, 1973) and Bayesian information criterion (BIC; Konishi et al., 2004). The lower the AIC or BIC, the better the model fits the data. The fit of nested models can also be examined using likelihood ratio tests (LRT; Vermunt et al., 1999; Giudici et al., 2000). If *p* < 0.05, the more general model shows significant improvements in fit than the constrained model at the .05 level.

Additionally, given a sequence of observations {*y*_*i,j*_} and a hidden Markov model, we could get the most probable sequence of the state estimates of {*S*_*i,j*_}, using the Viterbi algorithm (Viterbi, 1967; Forney, 1973; Rabiner, 1989). In the depmixS4 package, one can use the posterior() function to obtain the Viterbi most probable states, as well as the highest probabilities of a state sequence ending in a certain state at item *j* with all observations up to the item *j* taken into account.

## 4. Results

In this section, we first provide a description and visualization of the data, along with the HMM general results about state classifications and initial state modeling, followed by two sets of our transitions modeling questions: (1) modeling transitions between states in the two goal conditions; (2) modeling transitions based on accumulated correctness and confidence and their interactions.

### 4.1. Description of Data

**Figure 2:**
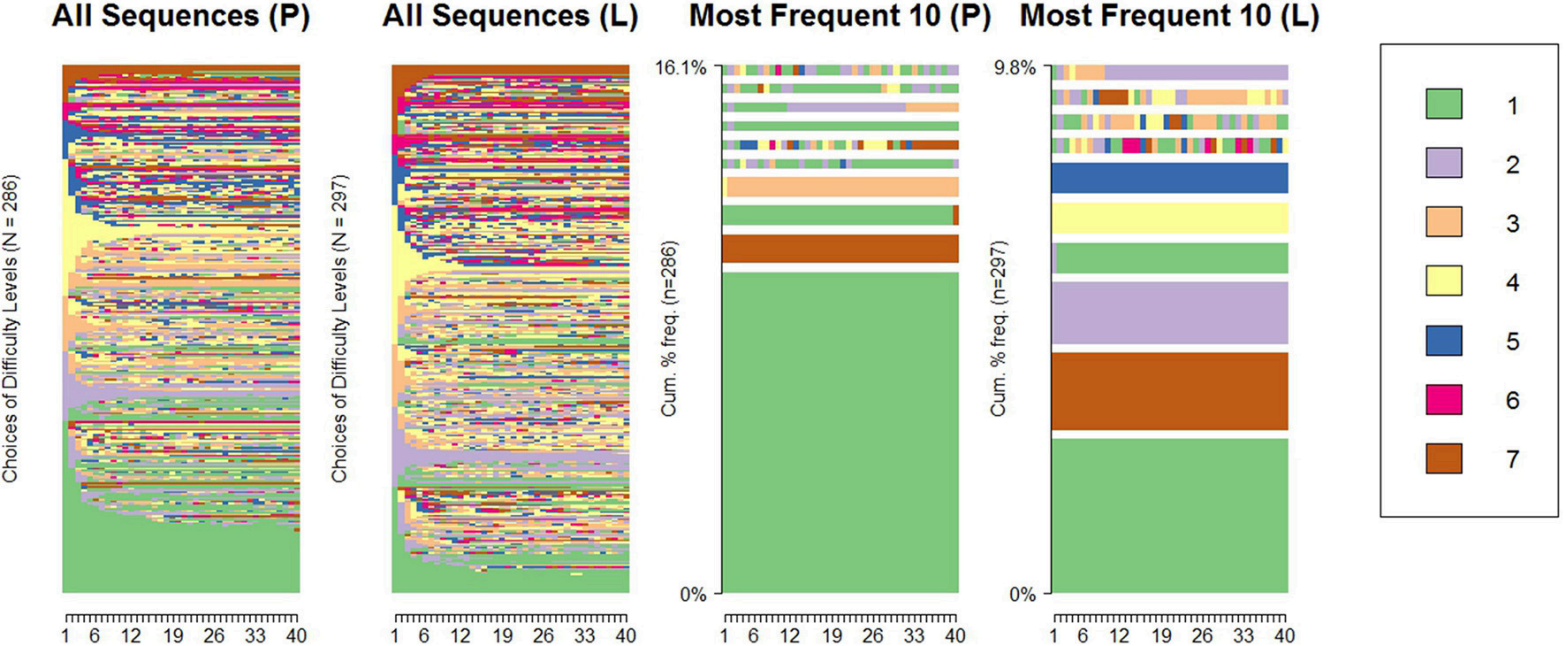
Sequences of difficulty choices and the most frequent 10 sequences across the performance (P) and learning (L) conditions.

Here we summarize the most relevant characteristics of the data. First we present the choice sequences and the visualization of the data: Figure 2 was created using the R package TraMineR (Gabadinho et al., 2011), and shows all the difficulty choice sequences and the ten most frequent sequences for the performance (P) and learning (L) goal conditions. The most frequent sequences are those with no transitions, where participants chose a level and stayed with it for the entire 40-item test, most frequently the extreme levels (level 1 and 7). Although there was not a clear difference between the conditions in the number or proportion of participants choosing to start and stay at the highest difficulty level (level 7; 3 participants in the performance goal condition, constituting 1.05%, and 5 in the learning goal condition, taking up 1.68%), substantially more participants chose to start at the lowest difficulty level (level 1) and stay there in the performance goal condition (33 or 11.54%) than in the learning goal condition (10 or 3.37%). In the learning goal condition there were also frequent sequences of starting and staying at level 2, 4, and 5 (as can be seen in the right-most panel), while in the performance goal condition these sequences were not frequent. Generally, there were also more switches in difficulty levels in the learning goal condition than in the performance goal condition. The average number of upward (i.e., from a lower manifest difficulty level to a higher one) and downward (i.e., from a higher manifest difficulty level to a lower one) transitions in the learning condition were 7.43 and 6.51, respectively, both slightly higher than in the performance condition (6.07 and 5.40, respectively).

**Figure 3:**
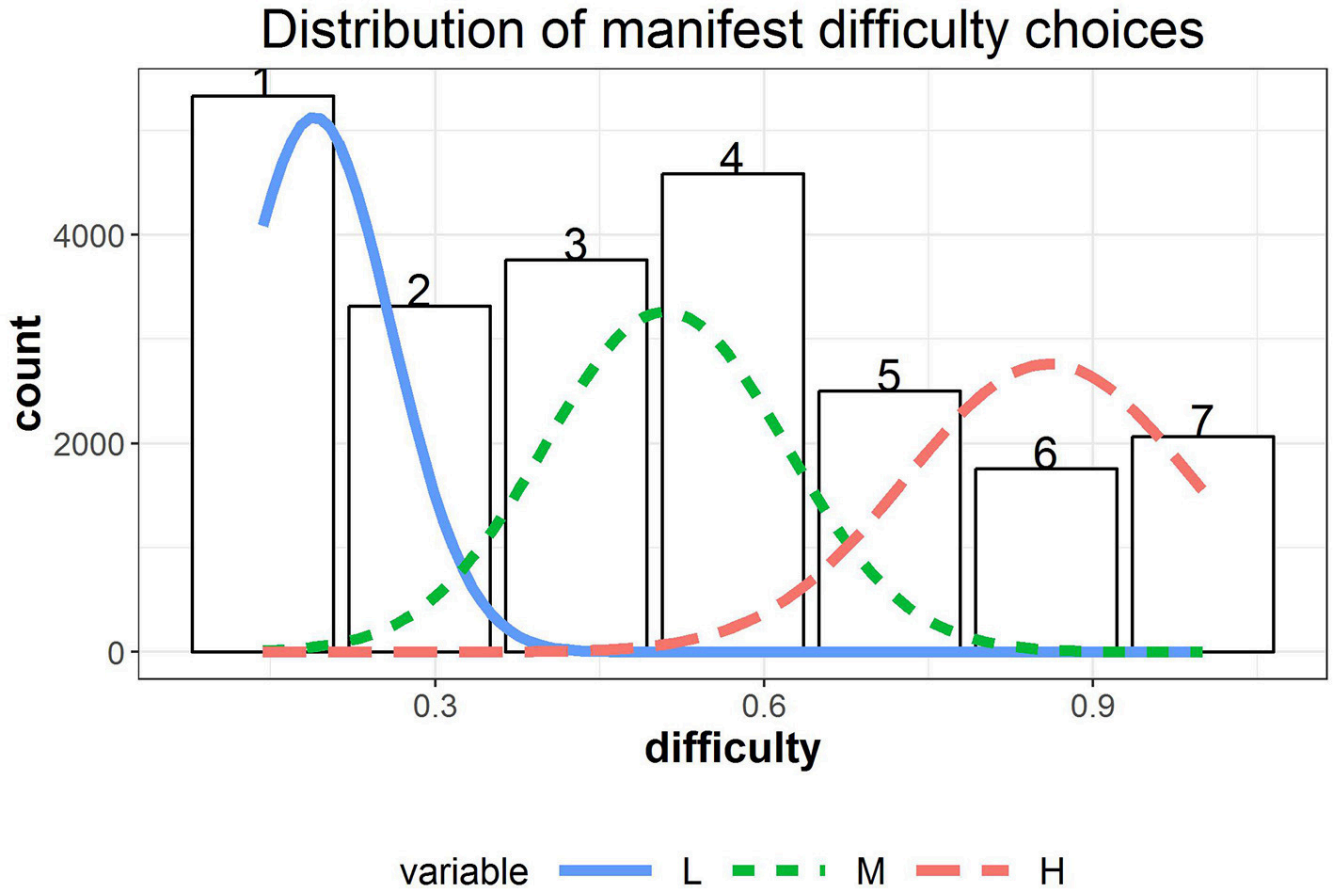
The distribution of manifest difficulty choices overlaid with the normal densities from the fitted 3-state HMM model.

Regarding the distribution of choices, among all chosen item difficulty levels (i.e., a total of 583 × 40 choices), 22.85% were at level 1, ranked as the highest proportion and followed by 19.67% at level 4, 16.13% at level 3, 14.22% at level 2, 10.73% at level 5, 8.87% at level 7, and 7.52% at level 6. The distribution of the manifest choices is displayed in Figure 3, which suggests that the marginal distribution of the data should follow a mixture distribution. The chosen item difficulty levels were negatively correlated with answer correctness (point-biserial correlation *r*_*pb*_ = − 0.30, *p* < 0.001) and perceived confidence (*r* = − 0.28, *p* < 0.001), while the latter two variables were positively correlated (*r*_*pb*_ = 0.60, *p* < 0.001).

**Figure 4:**
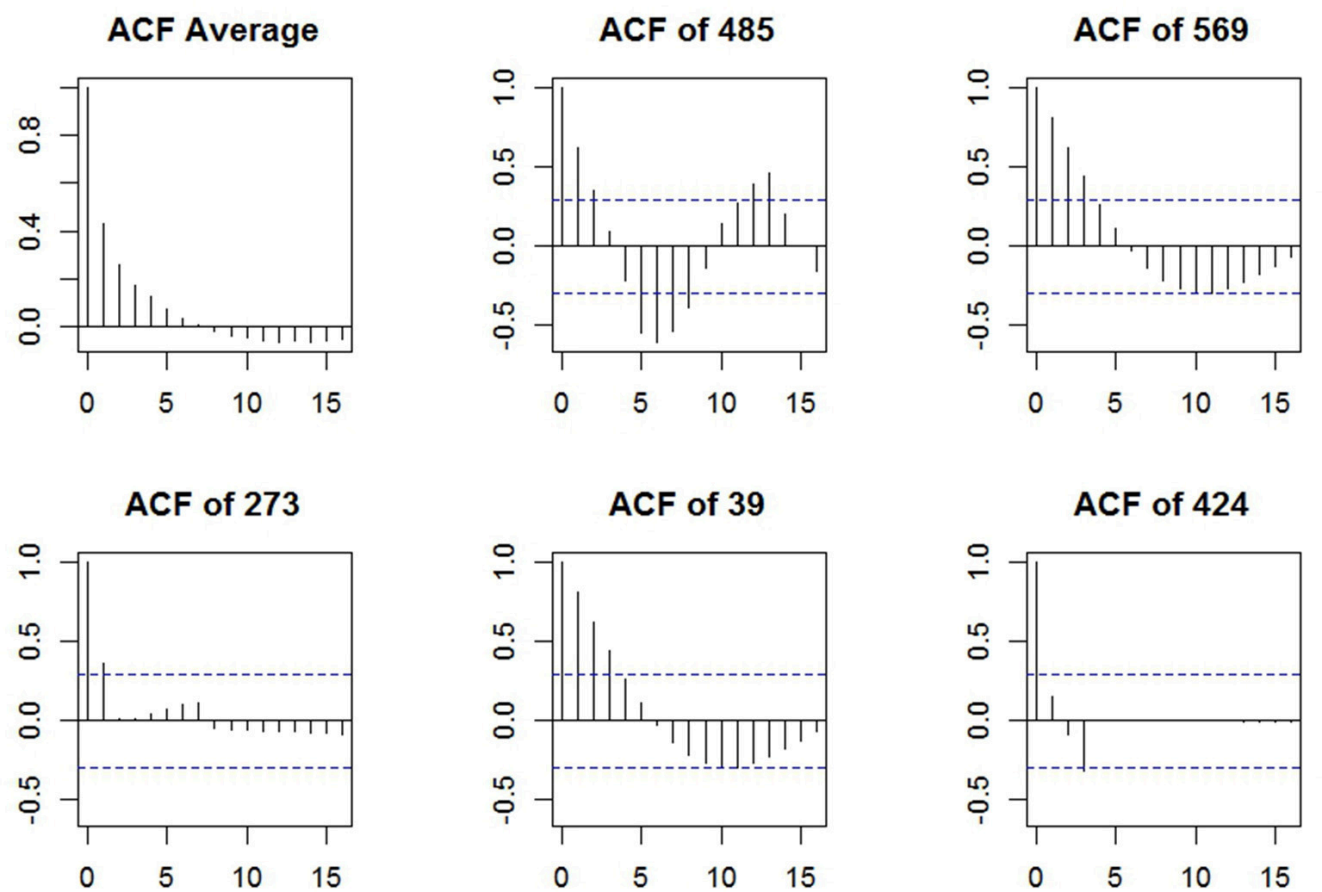
The autocorrelation functions in the residuals of manifest difficulty choices after removing participant and item effects; the dashed blue lines represent the 95% confidence limits.

To examine the item dependencies in the difficulty choices, we obtained the residuals of the manifest difficulty data after removing the participant and item effects in a generalized additive mixed model using the R package mgcv (Wood, 2006). The autocorrelation functions (ACFs) of the residuals are plotted in Figure 4 using the R package itsadug (van Rij et al., 2017), where the first panel displays the average ACF across participants, and the rest five are the ACFs for 5 randomly selected individuals. Although there were individual differences in the ACFs, on average the lag-1 autocorrelation was relatively high, around 0.44, suggesting the need of a first-order Markov model.

### 4.2. Hidden Markov Modeling Results

We used R package depmixS4 (Visser and Speekenbrink, 2010) to fit a series of HHM models to the data, which are summarized in Table 1. Comparison analyses indicated that a 3-state HMM (Model B; AIC = − 27223.54, BIC = − 27110.74) provided a better fit to the data than a 2-state HMM (Model A; AIC = − 16658.95, BIC = − 16602.55) based on the AIC and BIC (see Table 1). We hence present the results from 3-state HMMs. The parameter estimates of μ_*S*_*i,j*__ and σ_*S*_*i,j*__ in the measurement model are summarized in Table 1. Based on Table 1, the three latent states respectively represent low [L; μ_1_(σ_1_) = 0.19 (0.07)], medium [M; μ_2_(σ_2_) = 0.51 (0.12)], and high [H; μ_3_(σ_3_) = 0.86 (0.13)] item difficulty levels. The estimated normal densities are shown as overlaid on the manifest distribution in Figure 3. The fitted mixture distribution of the hidden Markov models was still able to capture the manifest distribution of the chosen difficulty levels.

**Figure 5:**
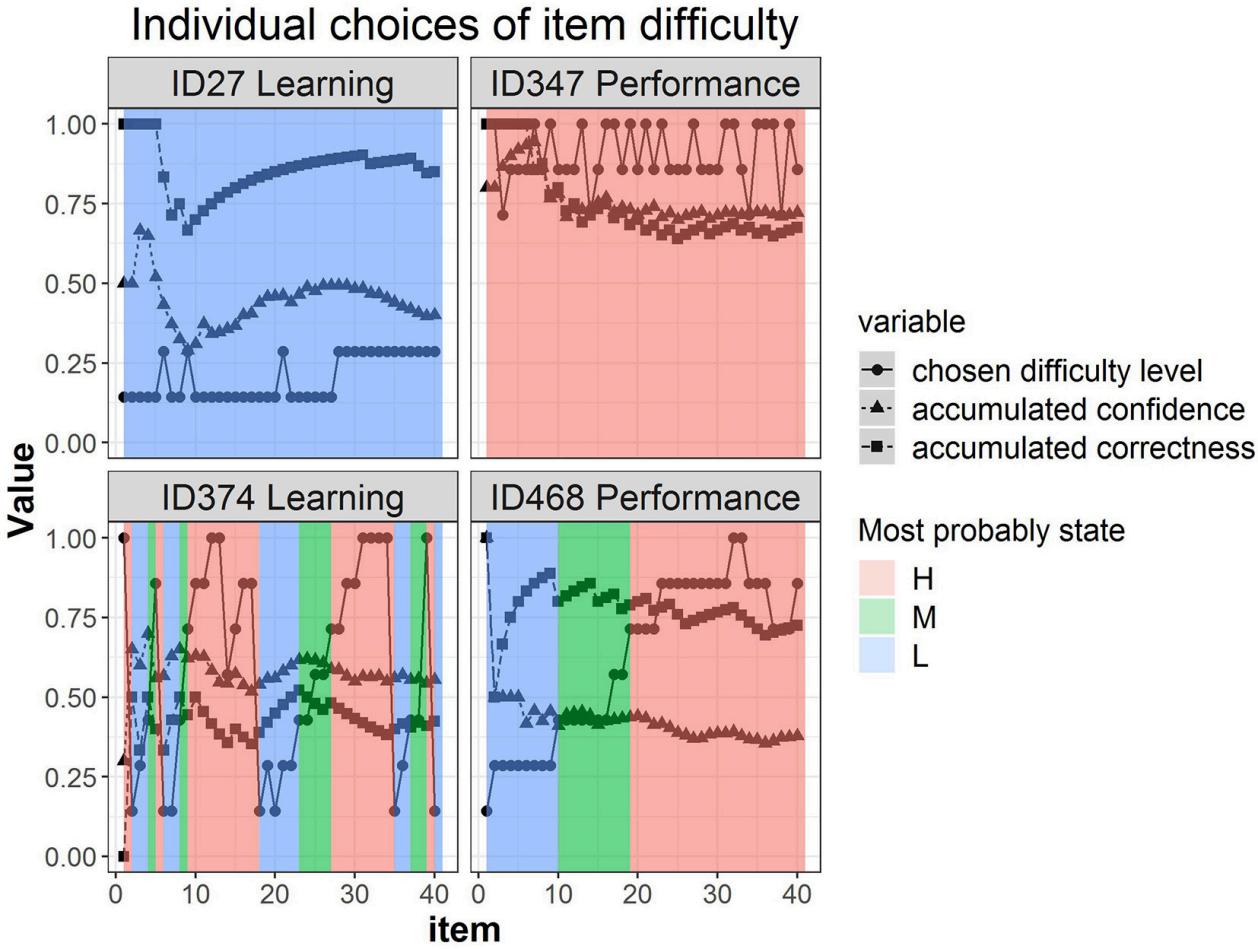
Four representative individuals' trajectories of item difficulty choices, accumulated confidence, and accumulated correctness, with the estimated most probable state at each item as identified by the 3-state hidden Markov model colored differently in the background.

Figure 5 shows four representative participants' trajectories of item difficulty choices, accumulated confidence, and accumulated correctness, accompanied by the estimated most probable state at each item colored differently in the background. For example, participant 27 in the learning goal condition stayed at the low-level difficulty across time (switching between level 1 and 2) and the most probable latent state throughout was the L latent class (background colored blue). The accumulated correctness was generally high (above 70%) and the accumulated confidence was relatively low (mostly below 50%), yet they co-varied across time. Participant 347 in the performance goal condition, on the other hand, chose high-difficulty items across time (levels 5, 6, and 7) and the most probable latent state was the H latent class (background colored pink). The levels of confidence and correctness for this participant were almost identical, with a decline at approximately item 8. Participants 374 and 468 showed more transitions in their choices of difficulty levels. Participant 468 showed a gradual increase in item difficulty choices reflected in the transition of the most probable latent state from L to M to H latent states (blue → green → pink) with a steady high accumulated correctness albeit moderately low accumulated confidence. Lastly, participant 374 showed many transitions upwards and downwards, while correctness and confidence were moderately low. Note that participant 374 provides an illustration of how the same manifest/observed difficulty level can be associated with different most probable latent states: level 4 (just above .5 on the y-axis) was linked to the H state when the surrounding difficulty choices were higher (between item 10 and 20), but linked to the M state when the preceding choices were lower (between item 25 and 30) (see arrows on the figure).

**Figure 6:**
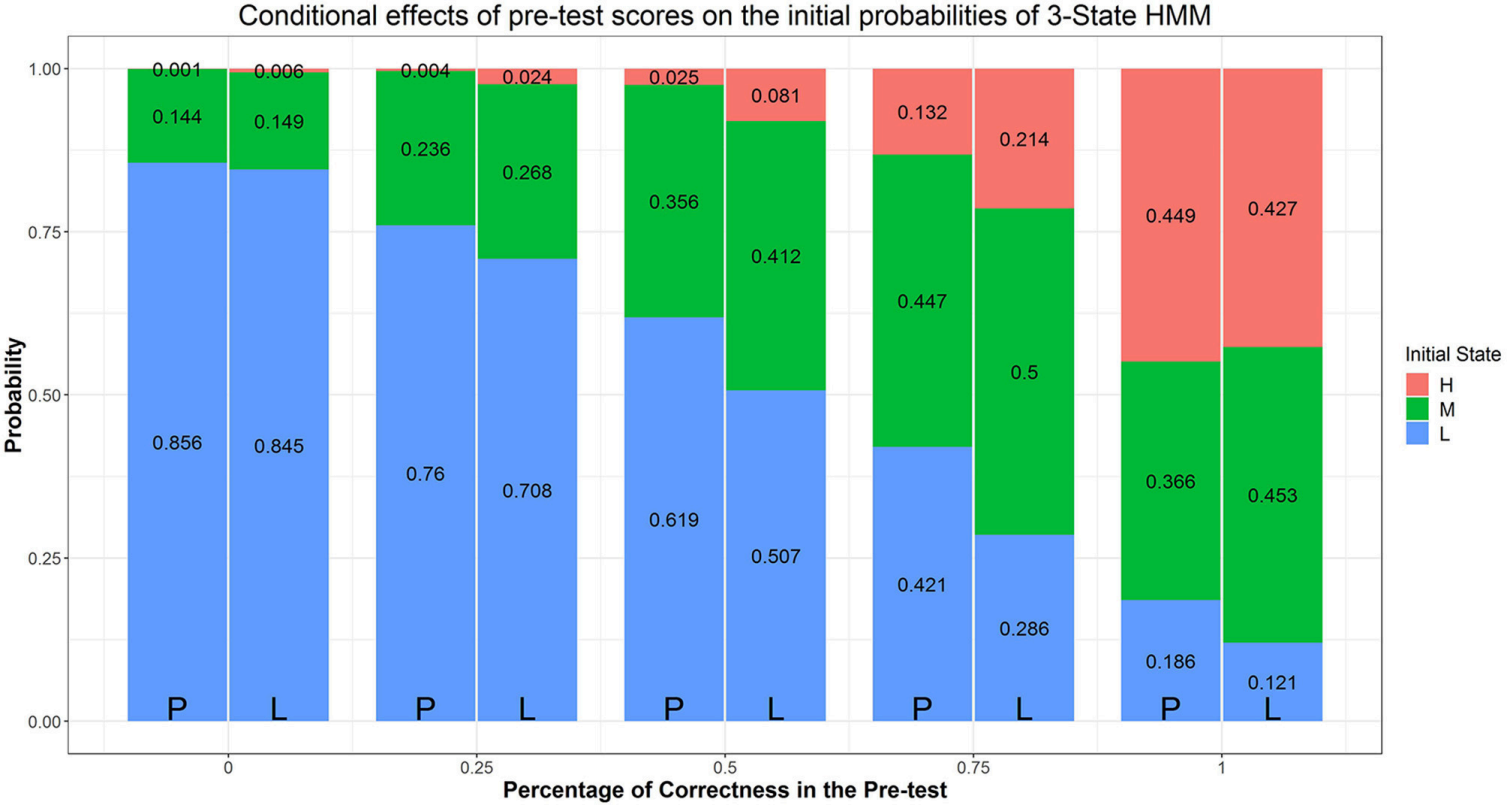
Effects of condition and prescore on initial class probabilities. The numbers in the stacked bars are expected probabilities evaluated at certain values of the predictors based on the model fitting results.

Similar to Arieli-Attali (2016) in predicting choices, we used pre-test score (i.e., percentage of correctness), goal condition, and their interaction as predictors of initial difficulty latent state; the resulting Model is Model B1. As noted above Arieli-Attali (2016) reported that test takers' selection of difficulty on the first item differed across goal conditions, with lower difficulty chosen in the performance group, after controlling for pre-test performance. Our model analysis adds to this finding by using the three latent states rather than manifest difficulty levels. Parameter estimates and fit indices are shown in Table 1. Model B1 fits significantly better than Model B based on the LRT ($\Delta_{\chi^{2}}$ = 71.26, Δ_*df*_ = 6, *p* < 0.05). As it is not intuitive for us to draw conclusions from the parameter estimates in the LO sense, we illustrate the logistic regression results in terms of expected probabilities evaluated at certain values of the predictors in stacked bar figures. Figure 6 indicate that when participants' pre-test scores are controlled, the expected probability of starting the test in a low-difficulty state compared to medium- or high-difficulty, is higher in the performance goal condition. Also it is evident from Figure 6, that within a condition, the higher the pre-test score, the higher the probability that the participant would initially be in a medium- or high-difficulty state. In particular, participants who answer fewer than half of the pre-test items correctly are more likely (above the 50%) to be in the low-difficulty initial state. Participants who have higher or full pre-test scores are more likely to be in initial state of medium- or high-difficulty. Now we turn to model transitions.

### 4.3. Research Question 1: Modeling Transitions in the Two Goal Conditions

**Figure 7:**
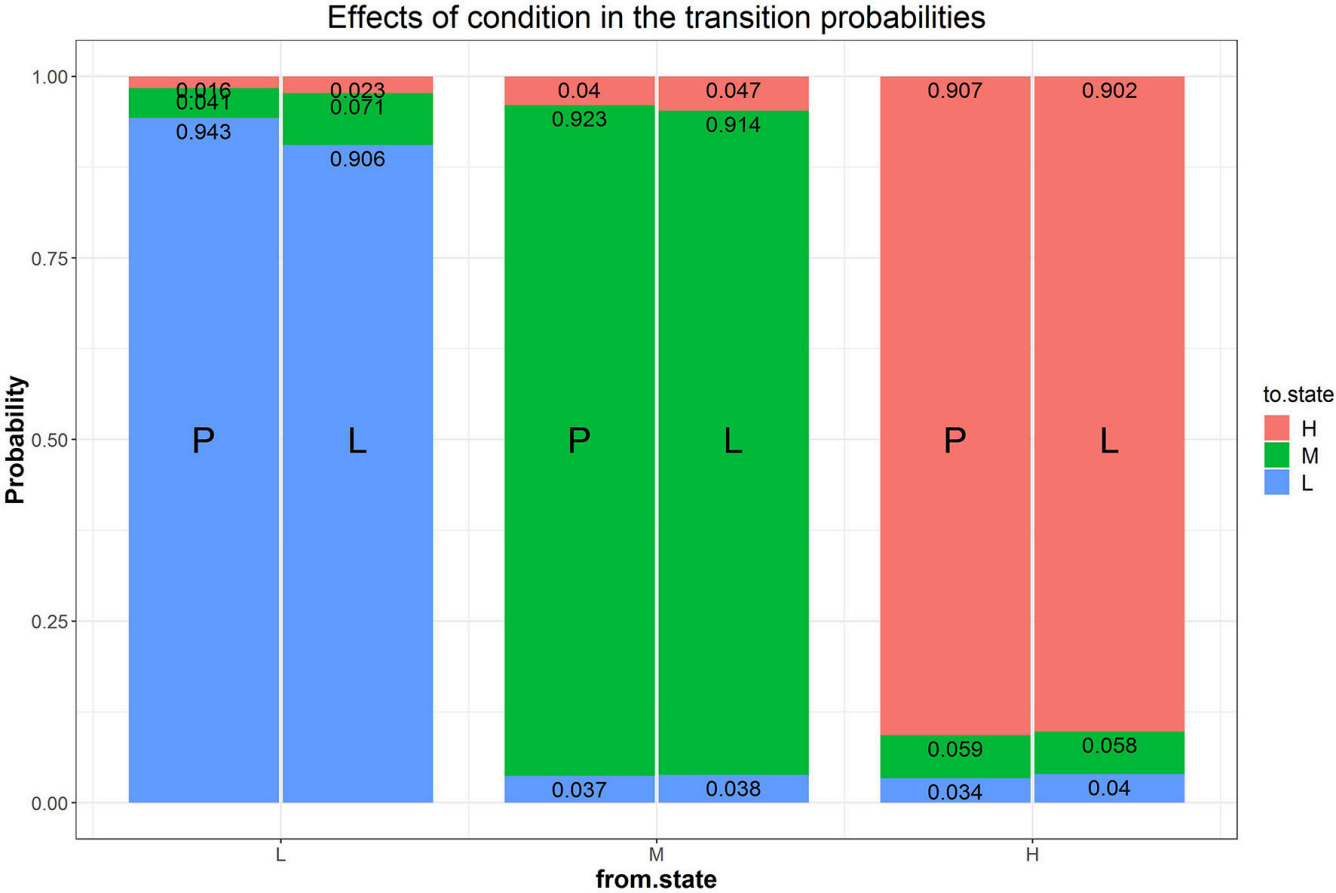
Effects of condition on the transition probabilities. The numbers in the stacked bars are expected probabilities evaluated at certain values of the predictors based on the model fitting results.

Our first research question addressed modeling transitions between states in the two goal conditions. We added a multinomial logistic regression of the transition probabilities with condition as predictor to Model B1 (i.e., Model B2a), which significantly improves the fit of Model B1 ($\Delta_{\chi^{2}}$ = 35.89, Δ_*df*_ = 6, *p* < 0.05) and has a lower AIC value^1^. Fitting results of Model B2a are presented in Table 1 and Figure 7. Figure 7 shows the expected probability of transitions to and from each of the three latent states separately for each condition. As this figure shows, in both conditions the most probable choice behavior is staying in the same latent difficulty state with probabilities of over 90% (recall that different manifest difficulty levels were included in each latent state). However, when looking at the transitions between conditions, the model predicts a higher likelihood of staying at low difficulty and a lower likelihood of upward transitions from low to medium difficulty in the performance goal condition. In other words, participants in the performance goal condition are expected to transition less from the low state, confirming and adding to the results reported by Arieli-Attali (2016) that test takers in the performance goal condition tended to choose the lower level more frequently than in the learning goal condition, shown here also when considering latent states and transitions between states. Note that transitions from the medium or high state (either upwards or downwards) were similar between the two goal conditions.

### 4.4. Research Question 2: Modeling Transitions Based on Correctness and Confidence

**Figure 8:**
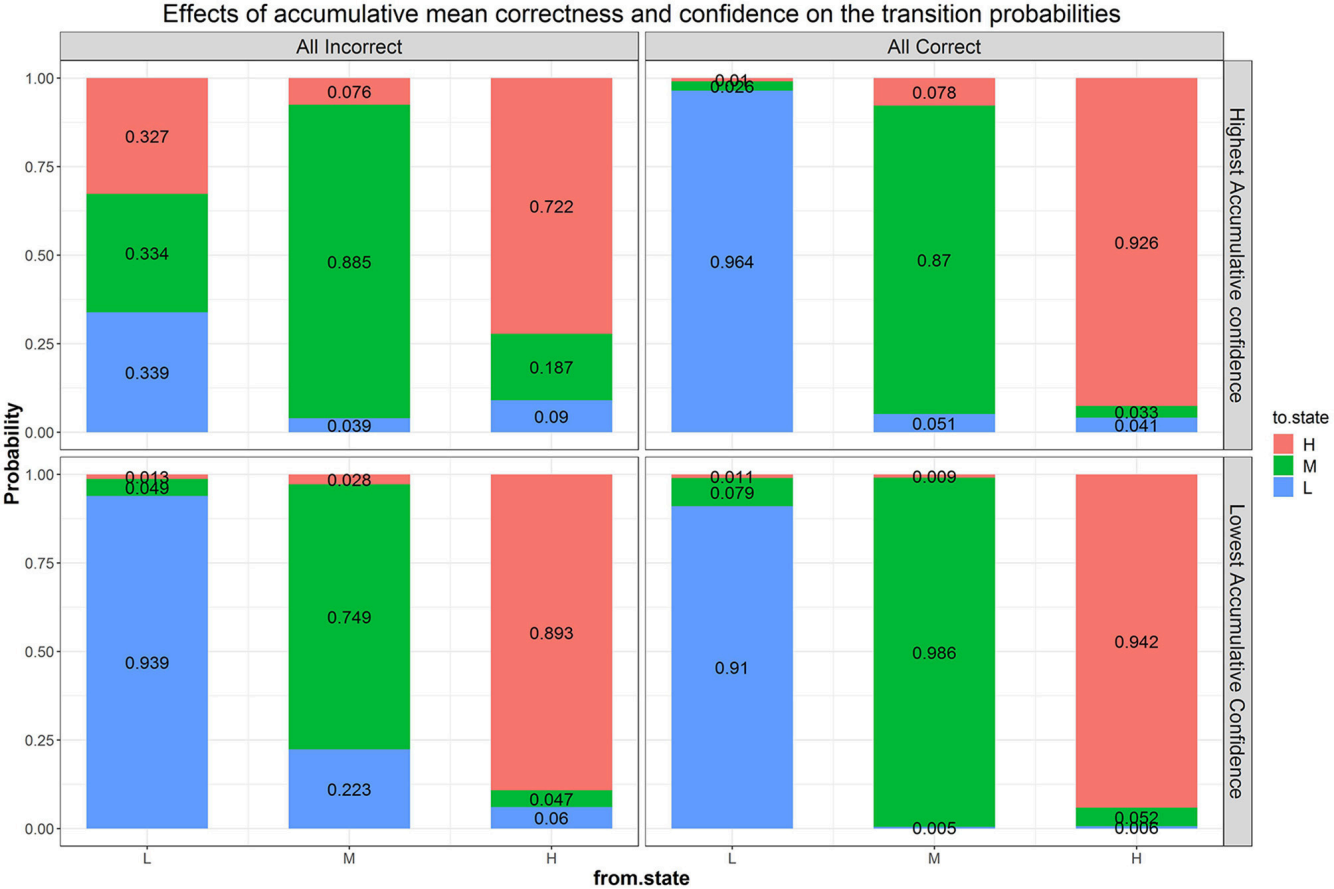
Effects of accumulated correctness, confidence, and their interactions on the transition probabilities. The numbers in the stacked bars are expected probabilities evaluated at certain values of the predictors based on the model fitting results.

**Figure 9:**
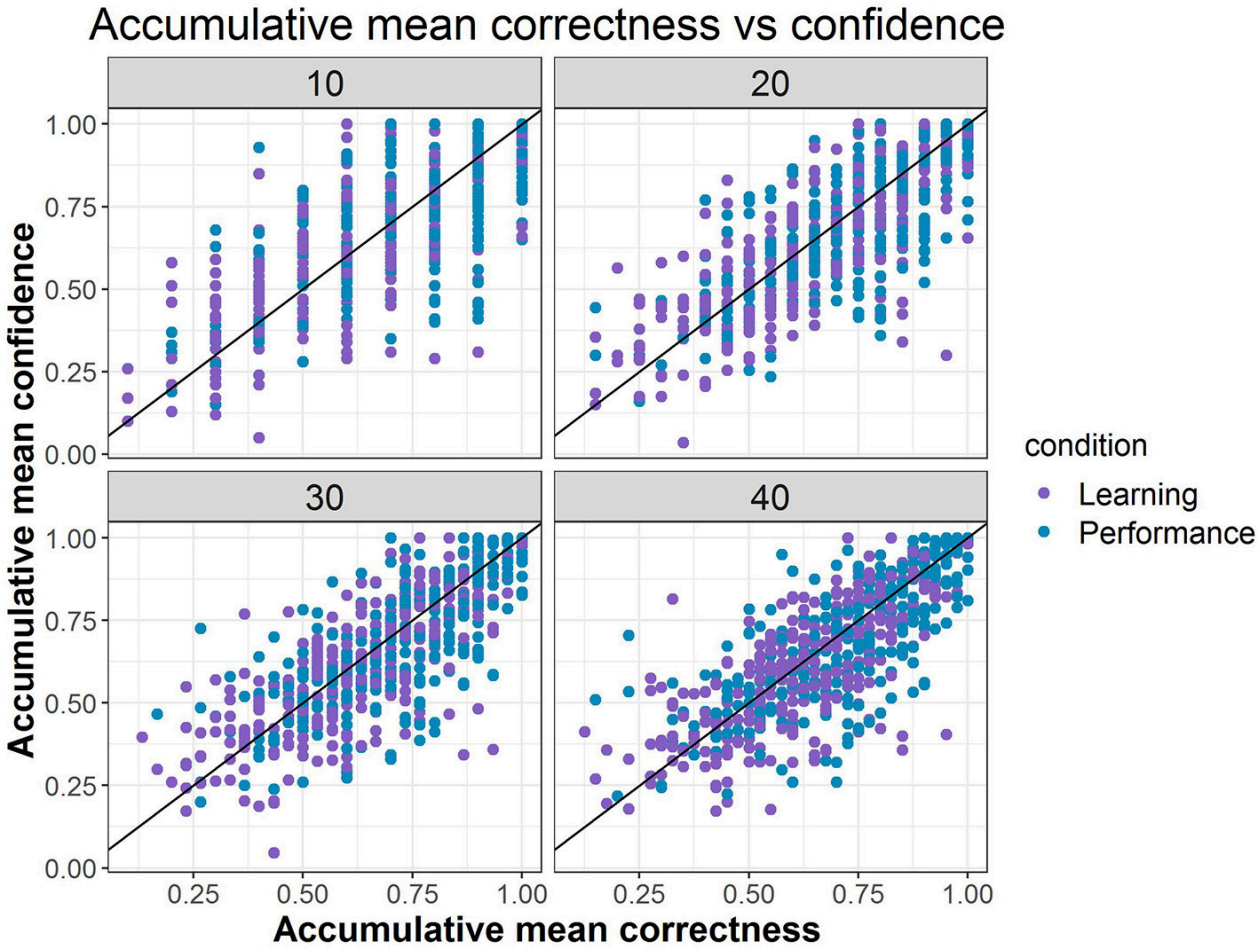
The distribution of accumulated confidence and correctness.

We next fitted a more general model than Model B1, with accumulated correctness and confidence across items as predictors without condition (i.e., Model B2b), to evaluate the influence of these characteristics on transitions. Parameter estimates and fit indices are presented in Table 1 and expected probabilities are displayed in Figure 8. Compared to Model B1, B2b fits the data significantly better ($\Delta_{\chi^{2}}$ = 219.83, Δ_*df*_ = 18, *p* < 0.05) and has lower AIC and BIC values. Note that the figure presents the four extreme quadrants of the two continuous scales. The horizontal line represents the accumulated correctness showing the extreme ends of the scale as “all incorrect” and “all correct” (from left to right), while the vertical line represents the accumulated confidence, showing the extremes of lowest and highest confidence (from bottom to top). As this figure shows, with high accumulated correctness (top and bottom right-side panels), expected probability of transitions is low and staying at the same difficulty state has the highest likelihood across the confidence scale. However, when accumulated correctness decreases (toward the quadrants in the top and bottom left-side panels) there is higher likelihood for transitions in both directions, and the likelihood of transitions increases as the confidence increases (i.e., illustrating the interaction between these factors). In particular, we can see expected downward transitions from the medium state when confidence is low (22.3%; bottom left-side panel), and from the high-state when confidence is high (27.7%; top left-side panel), as expected. However, we can also see that when the accumulated confidence is highest (top left-side panel; indicating over-confidence) participants are more likely to transition upwards from the low state (66.1%) equally to either the medium- or high-state. In other words, staying at the same state is the least probable in this case relative to other quadrants and states (recall that this quadrant is the extreme end of the confidence scale, and transition upwards from the low state are expected to increase as confidence increases). To get a sense of the frequency of participants with different relations between accumulated correctness and confidence, in particular considering the representation within each of the four quadrants illustrated in Figure 8, we show in Figure 9 the relation between accumulated correctness and confidence after 10, 20, 30, and 40 items. As can be seen, the data cluster along the diagonal increasingly as the number of items increased, with sparse representation in the quadrants with mis-matches between correctness and confidence. This suggests that test takers were overall well-calibrated in their confidence, with little representation of over- and under-confidence.

**Figure 10:**
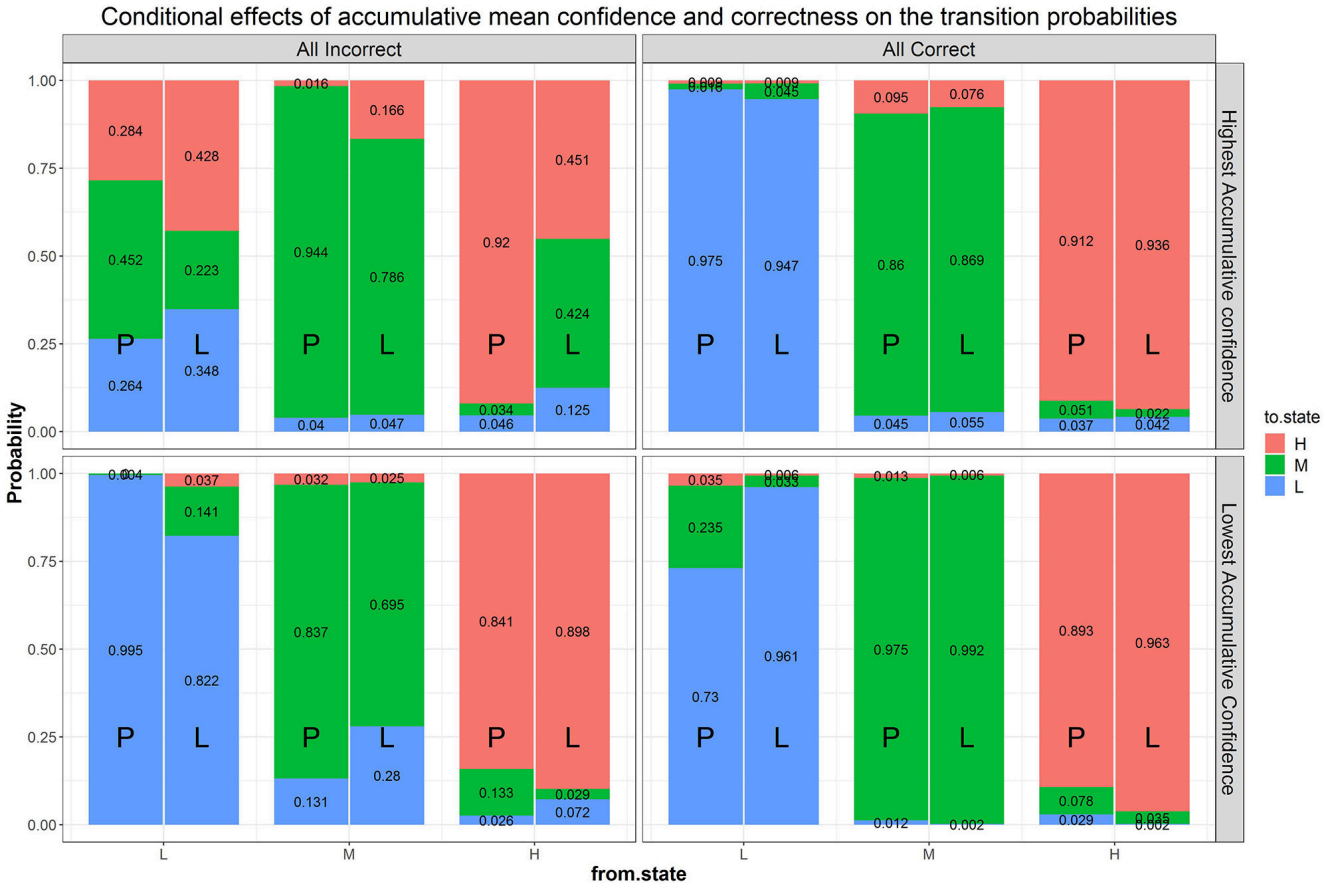
Effects of condition, correctness, confidence, and their interactions on the transition probabilities. The numbers in the stacked bars are expected probabilities evaluated at certain values of the predictors based on the model fitting results.

We then further added back goal condition as a predictor of the transition probabilities to Model B2b (i.e., Model B3), which significantly improved the fit of Model B2b ($\Delta_{\chi^{2}}$ = 68.67, Δ_*df*_ = 24, *p* < 0.05) and has a lower AIC value^2^. Figure 10 shows the same transition probabilities as in Figure 8 split by goal condition. The downward transitions when accumulated correctness decreases are also evident when split into the goal condition and are more so in the learning goal condition. The findings about higher likelihood of upward transitions in the over-confident quadrant are still evident when split into the goal conditions, with somewhat more transitions in the performance goal compared to learning goal condition (73.8 and 65.2%, respectively at the extreme quadrant of the confidence scale). A new finding from this split analysis is that there are also more transitions in the performance goal condition when accumulated correctness is high but confident is low (27%; bottom right-side panel, the quadrant indicating under-confidence).

## 5. Discussion

The purpose of our secondary data analysis from Arieli-Attali (2016) was to apply a hidden Markov model to test takers' choices of item difficulty in a self-adapted test. We investigated whether those choices could be modeled by the goal condition (learning vs. performance), as well as the test takers' correctness and confidence across items. Analysis of the data using the hidden Markov model identified three latent states of difficulty from the seven manifest levels. These three latent states correspond to low, medium and high difficulty levels, and may be an indication of a low, medium or high self-estimated ability and/or motivation. We first modeled test takers' initial difficulty state based on their pre-test scores and goal condition, confirming past results (Arieli-Attali, 2016) about preference of lower difficulty in the performance goal condition, showing it here also as a higher expected probability of starting in the low state in the performance goal condition after controlling for pre-test scores. The results here add to the understanding that this is not just the single first choice influenced by the goal orientation (in addition to the self-perceived ability), but rather it is the participant's latent state that is influenced and therefore drives the choices accordingly. This result further confirms that when the goal orientation is to excel at a task individuals may avoid taking on challenges (Dweck, 2006).

We then used the model to predict transitions across items, and found the highest likelihood was to remain at the same difficulty state across items. This is the main contribution of applying a latent state approach in this context, because manifested choices may show transitions attributable to random variability while actual latent states are less likely to change. When using only goal condition as a predictor, there was no difference in transitions from the middle- or high- states between the two goal conditions, however there was a slightly lower likelihood of upward transitions from the low state in the performance goal condition relative to the learning goal condition, confirming the overall finding that test takers in the performance goal condition applied a strategy of the “easy way out,” keeping low effort (Arieli-Attali, 2016).

The main contribution of this analysis is in the application of the HMM to model the interaction between answer correctness and confidence. We have shown that the likelihood of transitions increased when the accumulated correctness decreases. This result is intuitive as it means that participants were attentive to the correctness feedback and when they were overall wrong they tended to transition or change their metacognitive/motivational state. We found that downward transitions were more likely across the confidence scale as expected, but upward transitions were more likely when confidence increased for those who were in the low state, that is, we found that when confidence was highest, it reached the highest likelihood of about 2/3 upward transitions in the over-confidence end of the scale. This finding can be related to the literature on confidence and learning from errors by Metcalfe and colleagues (Butterfield and Metcalfe, 2001; Metcalfe and Xu, 2018). This line of research generally showed that people who made an error with high confidence were more likely to correct their mistake compared to a situation when the error was made with low confidence (the hypercorrection phenomenon). One of the explanations of this phenomenon is the surprise/attention explanation, which says that individuals experience surprise at being wrong when they were sure they were right, and as a consequence they rally their attentional resources (Butterfield and Metcalfe, 2006; Metcalfe et al., 2012). In our study we showed that individuals with high confidence who were proven incorrect were more likely to change difficulty state as reflected in more transitions upwards. The transitions upwards may be a reflection of being more attentive or putting forth more effort, similar to what occur under the hypercorrection phenomenon.

We also found that accumulated correctness and confidence interacted with goal condition in predicting transitions. The transitions when accumulated correctness decreases were also likely when split into the goal conditions but the downward transitions have higher likelihood in the learning goal condition, while the upward transitions in the over-confidence case have higher likelihood in the performance goal condition. This analysis also revealed a new finding of higher likelihood of upward transitions in the performance goal condition when accumulated correctness was high but confident was low, i.e., in the under-confidence end of the scale. These two findings together, that in the performance goal condition test takers were more likely to transition upwards from the low state in both mis-matched conditions (over- and under- confidence), indicate the specific interaction of the goal with correctness and confidence, and may suggest that when (1) participants are instructed to do their best, (2) they experience mis-match between what they think they know and what they actually know (feedback of correctness), and (3) they are in the low state without possible downward transition, they try to “find their luck” someplace else or decide to put more effort. This finding may suggest that mis-calibration between confidence and correctness could serve as a motivating factor, as being in the low state in the performance goal condition has been shown to stem from low motivation (Arieli-Attali, 2016). This combined pattern was not found for the learning goal condition, suggesting that evidence about mis-calibration when one is striving to learn has less of an effect (i.e., it had an effect in over-confidence, but not in under-confidence).

These results are consistent with the literature on goal orientation, showing that participants who are encouraged to use the test for learning rather than focusing on performance are more likely to seek challenges and show resilience amid difficulties (Yeager and Dweck, 2012). However, our additional findings about the interaction between correctness, confidence, and goal orientation further shed light on the complexity of the choices made in self-adapted test. The interactions we found suggest that the test takers' goal (i.e., whether the participant needs to maximize one's score, as the goal of the test), confidence across items (as a reflection of one's internal states), and correctness (as an outside feedback) together may form a recursive feedback loop that results in the changes of an individual's motivational and/or metacognitive state and further affects choice behavior.

To summarize, in this study we explored ways to learn about the motivation and feeling of knowledge of test takers and its affect on their actions while engaging in an interactive self-adapted test, via analyzing process data. Motivation and engagement is particularly crucial in low stakes assessment programs (such as the National Assessment of Educational Progress program, or the Trends in International Mathematics and Science Study), where test scores have no personal consequences for individuals, potentially resulting in low motivation to do one's best, and subsequently threatening the validity of the test scores. While low stakes programs make attempts to make their tests more interactive and appealing to participants in order to increase their engagement, we offer insights on how goal orientation, correctness and confidence influence choices that determine the course of the test. More research is needed to learn about how complex choice making in simulation- and game-based assessment can be modeled by factors inherent to the simulation or the game (such as curiosity, challenge seeking, sense of satisfaction, and the like).

## Author Contributions

MA-A: research design, data interpretation, and paper writing; LO: data analysis and interpretation, and paper writing; VS: data interpretation and paper writing.

### Conflict of Interest Statement

The authors declare that the research was conducted in the absence of any commercial or financial relationships that could be construed as a potential conflict of interest.

## Footnotes

^1^Please note that the BIC of B2a is larger than that of B1.

^2^Please note that the BIC of B3 is larger than that of B2b.
